# Ligands as a universal molecular toolkit in synthesis and assembly of semiconductor nanocrystals

**DOI:** 10.1039/c9sc05200c

**Published:** 2020-02-10

**Authors:** Hyeonjun Lee, Da-Eun Yoon, Sungjun Koh, Moon Sung Kang, Jaehoon Lim, Doh C. Lee

**Affiliations:** a Department of Chemical and Biomolecular Engineering , KAIST Institute for the Nanocentury , Korea Advanced Institute of Science and Technology (KAIST) , Daejeon 34141 , Republic of Korea . Email: dclee@kaist.edu; b Department of Chemical and Biomolecular Engineering , Sogang University , Seoul 04107 , Republic of Korea; c Department of Energy Science , Center for Artificial Atoms , Sungkyunkwan University (SKKU) , Suwon , Gyeonggi-do 16419 , Republic of Korea . Email: j.lim@skku.edu

## Abstract

Successful exploitation of semiconductor nanocrystals (NCs) in commercial products is due to the remarkable progress in the wet-chemical synthesis and controlled assembly of NCs. Central to the cadence of this progress is the ability to understand how NC growth and assembly can be controlled kinetically and thermodynamically. The arrested precipitation strategy offers a wide opportunity for materials selection, size uniformity, and morphology control. In this colloidal approach, capping ligands play an instrumental role in determining growth parameters and inter-NC interactions. The impetus for exquisite control over the size and shape of NCs and orientation of NCs in an ensemble has called for the use of two or more types of ligands in the system. In multiple ligand approaches, ligands with different functionalities confer extended tunability, hinting at the possibility of atomic-precision growth and long-range ordering of desired superlattices. Here, we highlight the progress in understanding the roles of ligands in size and shape control and assembly of NCs. We discuss the implication of the advances in the context of optoelectronic applications.

## Abstract 2

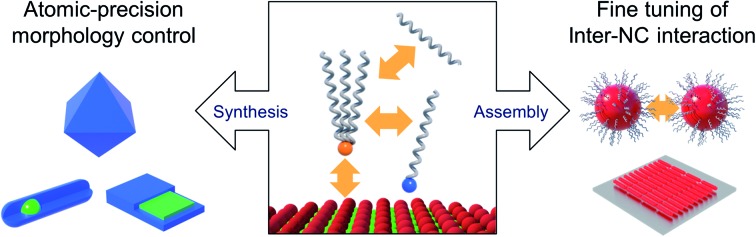
The multiple ligands with different functionalities enable atomic-precision control of NCs morphology and subtle inter-NC interactions, which paves the way for novel optoelectronic applications.

## Introduction

Colloidal semiconductor nanocrystals (NCs) hold the promise of important advances in diverse applications such as displays,^1^ solid-state lighting,^2^ lasing^3^ and photovoltaic energy harvesting.^4^ Much progress has been made in understanding the chemistry and developing general routes to synthesize certain classes of semiconductor NCs. In fact, the recent launch of flat panel displays based on semiconductor NCs has been a pinnacle of progress, which makes their usefulness more than a mere hand-waving notion of technological possibility. The future technological progress will certainly depend on understanding of the applicable chemistry to create NCs made of useful materials. Equally important is detailed knowledge of the physical interaction of NCs with themselves or surroundings which influences the assembly behavior of NCs in liquid and solid phases. Surface ligands on colloidal NCs have unambiguously significant bearing on the growth chemistry and inter-particle forces.

**Fig. 1:**
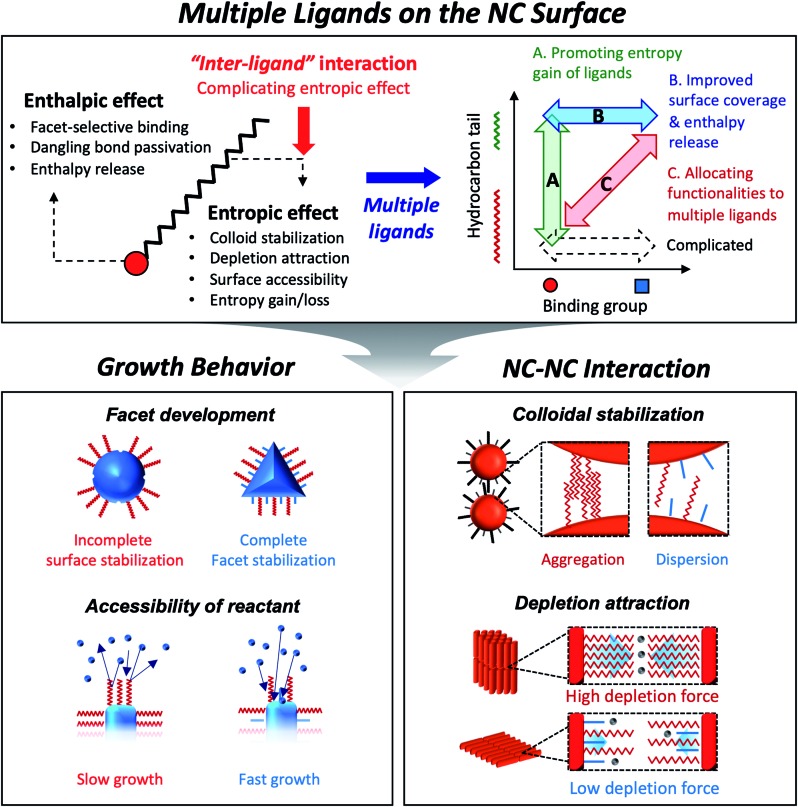
Schematic illustration on the role of surface ligands and benefits of multiple ligands in design of NC synthesis and assembly.

Capping ligands used in the conventional synthesis of colloidal NCs consist of polar head groups and aliphatic chains (upper panel in Fig. 1). The binding moiety of ligands adsorbs to the surface atoms of NC, leaving hydrocarbon chains extended into the solution to form a steric barrier. The interaction between ligands and the NC surface has been key to achieving uniform NCs, even in large-quantity synthesis.^5^ In fact, in earlier studies, uniform NCs were possible only by time- and labor-consuming size selection processes.^6^ Present synthetic protocols utilize various surface ligands to promote the size-focusing condition in the course of reaction that is recently capable of achieving minimal size dispersity less than 5%.^7^ The aliphatic chains in capping ligands confer the colloidal stability of NCs: ligand–solvent and ligand–ligand interactions are expressed in enthalpic and entropic terms in Gibbs free energy of mixing^8^ and make NCs disperse in an appropriate medium.

In the wet chemical synthesis of NCs, increasing attention has turned to the design of NCs in various shapes, compositions, and heterostructures. The thrust of research has extended to three levels of sophistication: (i) control of NC shape and heterostructures in atomic precision; (ii) facet-selective passivation of surface reaction; and (iii) modulation of inter-NC interactions in solution and the film of NCs. Understanding ligand-complex and ligand–surface interactions enabled high-precision heteroepitaxy in the growth of NCs, opening an avenue for tuning electronic structures of heterostructured NCs, such as spatial partitioning of carriers^9^–^11^ or strain-induced modification of exciton fine structures.^12^,^13^ Several studies have unraveled facet-specific binding of different types of ligands^14^,^15^ (*e.g.*, X-, L-, or Z-type ligands classified by electron donating or accepting characters) that passivate surface dangling bonds chemically^16^ or electronically.^17^ In addition, long-range ordering of NCs has become possible with the growing understanding on interparticle interactions modulated with surface ligands. Such orientation takes more complicated sets of interactions in the case of anisotropic NCs, such as nanorods (NRs) or nanoplatelets (NPLs).

In choosing surface ligands for NC synthesis, one may likely take rule-of-thumb guidelines on the binding nature of ligands, for example, alkylphosphonic acids as a trigger of anisotropic growth. Such an empirical strategy can be corroborated with simplified computational modeling that assumes the ligand as a binding group terminated with a hydrogen or methyl group.^18^ However, recent computational studies suggest that the hydrocarbon counterpart makes an equally, if not more, significant contribution to the development of NC shape and assembled structures. The beauty of the advanced simulation approaches is the cross-examination between simplified models and experimental observation in various cases. For instance, the steric hindrance of hydrocarbon chains also influences surface energy-driven facet development because the enthalpy release associated with surface binding of ligands is largely compensated by entropy penalty associated with limited conformational freedom.^19^,^20^ A closely packed ligand shell on NCs affects the permeability of the reactant to the surface of NCs which determines the growth rate of the facet. Unexpectedly poor or enhanced solubility of the NC dispersion or directed self-assembly of NCs is understood as a contribution from freezing and melting of hydrocarbon chain bundles and the corresponding inter-NC interaction.

As explained above, both a binding head group and a hydrocarbon tail play crucial roles in the entire sequence from the development of NCs to their macroscopic assembly. And it implies that, for engineering NCs in a precise manner, the binding head group and the hydrocarbon tail in ligands need to be independently controlled from each other to avoid redundancy of functions. Such necessity calls for the introduction of two or more ligands, namely, multiple ligands. Multiple ligands are responsible for allocating complex functionalities of a single ligand to their different moieties. For example, it is possible to promote the colloidal stability of NCs by employing multiple ligands with the same binding group but different hydrocarbon tails, which prevent entropy loss by inter-ligand interaction (case A in the upper panel in Fig. 1, see entropic ligands in the second chapter.). In spite of reduced colloidal stability, introduction of two or more short ligands with different binding groups is beneficial to improve the degree of surface passivation, as demonstrated in NC-based photovoltaic research (case B in the upper panel in Fig. 1).^17^ Even if we adopt short-chain ligands with different binding modes into an incompletely passivated NC surface with long-chain ligands, we are able to accomplish enhanced surface passivation without disrupting the colloidal stability of NCs (case C in the upper panel in Fig. 1, see the co-passivation strategy in the first chapter). These examples are hardly achievable with a single ligand-based approach.

In this review, we highlight the progress in the growth and assembly of semiconductor NCs based on accumulating knowledge of ligands. In particular, we discuss and outline a line of work demonstrating that the multiple ligand system can serve as a universal molecular toolkit. In other words, atomic and molecular-level control on growth and assembly of semiconductor NCs has been manifested in ligands giving rise to development of facets on NCs, anisotropic growth of NCs by controlling relative reactivity for different facets, and modulation of inter-NC interaction (lower panel in Fig. 1). The multiple ligand approach offers a new opportunity as it presents more exquisite and elaborate control in synthesis and assembly of NCs. Our discussion aims to underline the importance of ligands in colloidal semiconductor NCs in the context of optoelectronic applications, with the focal point on light-emitting devices.

## Ligands in morphology control of NCs

The past three decades have seen remarkable progress in wet chemical growth of colloidal semiconductor NCs, paving the way for large-quantity synthesis of monodisperse and shape-controlled semiconductor NCs. It has turned out that the size and shape of NCs are directly linked to their photophysical properties, characterized and analyzed by advanced spectroscopy and computational studies. Besides the obvious relationship between energy gap and NC size in the quantum confinement regime, NCs with prolate geometry can exhibit wider energy spacing between bright and dark exciton states than spherical NCs.^21^,^22^ 1-dimensional NRs exhibit unique optical anisotropy along the elongated direction for both absorption and emission.^23^,^24^ Their polytypic growth, such as a tetrapod, on seed NCs shows a significantly higher absorption cross section because of the antenna effect by four 1-dimensional arms.^25^ The structure–property relationship provides the impetus for the synthesis of NCs in atomic scale precision^16^,^26^,^27^ or in high homogeneity of size and shape.^28^,^29^

**Fig. 2:**
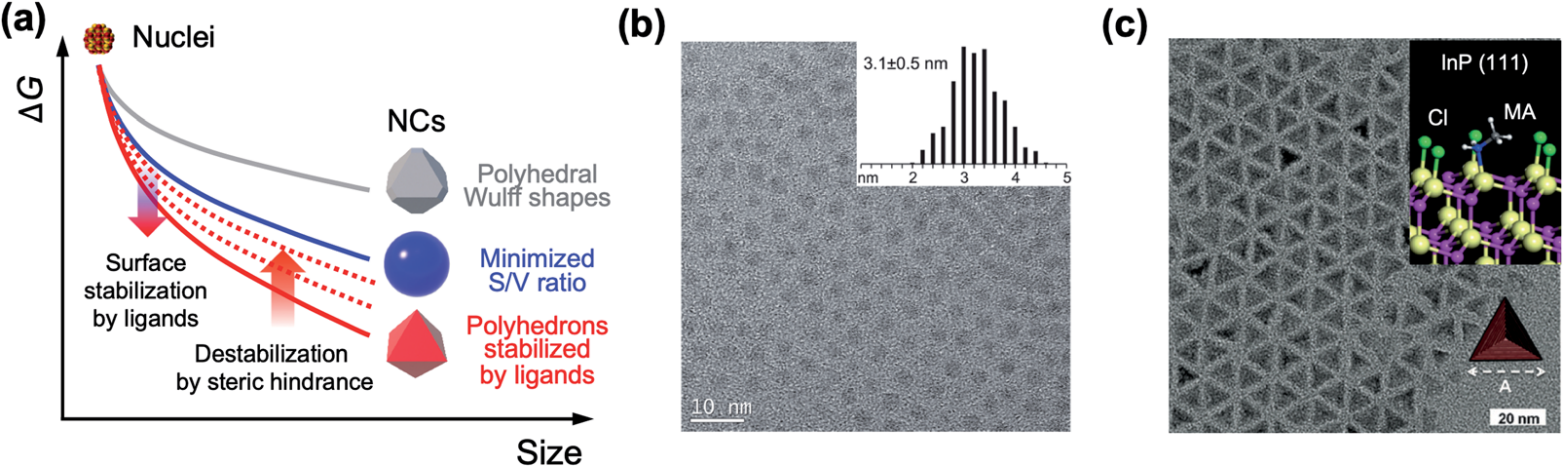
(a) Schematic illustration on the change in the free energy of NCs as a function of size. In realistic synthesis (denoted as a blue solid line), thermodynamic energy minimization drives NC morphology from Wulff polymorphs (denoted as a grey line) to highly truncated or spherical shape. If surface ligands sufficiently stabilize specific facets, the morphology of NCs transforms to faceted NCs (red line). However, steric hindrance between surface ligands increases the surface energy and is likely to truncate faceted NCs (from red solid to red dotted lines). (b) Spherical InP NCs synthesized with singular myristate ligands at 400 °C. Reproduced with permission from ref. 31. Copyright 2015 American Chemical Society. (c) Illustration of the co-passivated InP (111) facet with chloride (green) and methylamine (MA, white and grey) (inset) and a TEM image of the tetrahedral InP NCs. Reproduced with permission from ref. 16. Copyright 2016 Wiley-VCH.

Arrested precipitation has proven to be a powerful approach for colloidal growth of semiconductor NCs. In this wet chemical synthesis protocol, capping ligands passivate the surface of NCs in dynamic equilibrium during the growth. Without capping ligands, only the formation energy described in volume and surface terms would account for the growth of crystals. For instance, NCs in several-nm regime grow into near-spherical geometry rather than Wulff shapes, because a low surface-to-volume ratio results in low Gibbs free energy outweighing the adverse effect of high index facets (from a grey to a blue line in Fig. 2a).^19^ In the presence of surface ligands, the growth often takes complicated yet controllable routes. Under typical synthetic conditions, semiconductor NCs are terminated with polar and nonpolar facets (*e.g.*, polar (0001) and (0001[combining macron]) facets and the nonpolar (112[combining macron]0) facet for wurtzite CdSe) where the surface energy varies by an order of magnitude (*e.g*., ∼40 meV Å^–2^ for the nonpolar (112[combining macron]0) facet *versus* 180 meV Å^–2^ for the polar (0001) facet of CdSe).^18^ Surface ligands interact with surface atoms by donating one electron (X-type ligands like thiolates, carboxylates or phosphonates), by transferring two electrons (L-type ligands like alkylamines) to surface atoms, or by accepting two electrons from surface atoms (Z-type ligands such as metal carboxylates). Such a ligand–surface interaction could lower the surface energy of NCs (*e.g.*, 10s meV Å^–2^ for CdSe). Equally important is the ligand–ligand interaction: on a fully passivated facet covered with dense surface ligand layers, limited conformational freedom between hydrocarbon chains leads to a higher surface energy.^19^ In case the steric effect of ligands increases the surface energy beyond what bare facets would possess, the NCs could expose nonpolar and ligand-free facets, likely resulting in truncated polyhedral morphology, as demonstrated by size-dependent shape evolution of PbS NCs from octahedral to truncated cubic morphology.^26^ The competition between surface-stabilizing ligand–surface interaction and surface-destabilizing ligand–ligand interaction is responsible for the growth of non-spherical NCs and makes the full passivation of the specific facet difficult in the wide range of NC size.

Because most surface ligands used in the synthesis of semiconductor NCs have long chains (*e.g.*, number of carbons varying from twelve to eighteen) and are likely to exhibit large entropic penalty when they are packed, attaining fully passivated semiconductor NCs with polyhedral morphologies seems challenging. However, a recent study shows that the combination of atomic and conventional surface ligands can mitigate the entropic penalty caused by ligand layers. Jeong and co-workers demonstrated that passivation of InP NCs with chloride and primary amine develops highly monodisperse tetrahedron morphology terminated with four (111) facets.^16^ In terms of notation of fractional dangling bonds, surface atoms of InP NCs are terminated with dangling electrons, 0.75 and 1.25 for In and P, respectively.^16^,^30^ Different from typical spherical InP NCs produced in the presence of singular carboxylate ligands at a high reaction temperature (Fig. 2b),^31^ InP NCs passivated with chloride and oleylamine develop sharp (111)-facets at a moderate temperature (Fig. 2c) as a result of surface energy minimization (from a grey to a red line in Fig. 2a). X-ray photoelectron spectroscopy and theoretical calculation reveal that the tetrahedral InP NCs result from four In-terminated (111) facets stabilized by three chlorides and one primary amine per a (2 × 2) unit (Fig. 2c, upper right inset). The understanding offers an unprecedented avenue to remarkable monodispersity in the size and shape of non-spherical InP NCs, and this became possible with the knowledge of ligand interactions.

Semiconductor NCs grow into anisotropic morphology when anisotropy exists in the surface energy of facets. In the cases of II–VI semiconductor NCs, a long list of literature studies has reported the synthesis of anisotropic NCs and anisotropic heterostructures (*e.g.*, 1-dimensional NRs,^32^–^34^ 2-dimensional NPLs,^35^–^37^ branched NCs,^38^–^40^ *etc.*). In the growth, crystal structures of II–VI semiconductors, zinc blende and wurtzite, come into play. In the hexagonal wurtzite structure, the (0001) and (0001[combining macron]) facets exhibit higher surface energy than other facets like (101[combining macron]0) and (1120) because there are no counter ions neutralizing the outmost atoms (so-called, polar surface). In addition to such asymmetric nature, specific surface ligands (*e.g.*, alkylphosphonic acids) bind more strongly to the (0001) and (1120) facets than to the (0001[combining macron]) facet and promote the anisotropic growth along the [0001[combining macron]] direction.^14^,^41^ One must identify reaction conditions to achieve the desired crystalline phase^42^ and to promote kinetically controlled growth^38^ (*i.e.*, reaction is governed by surface reactivity, not by the diffusion flux of monomers).

**Fig. 3:**
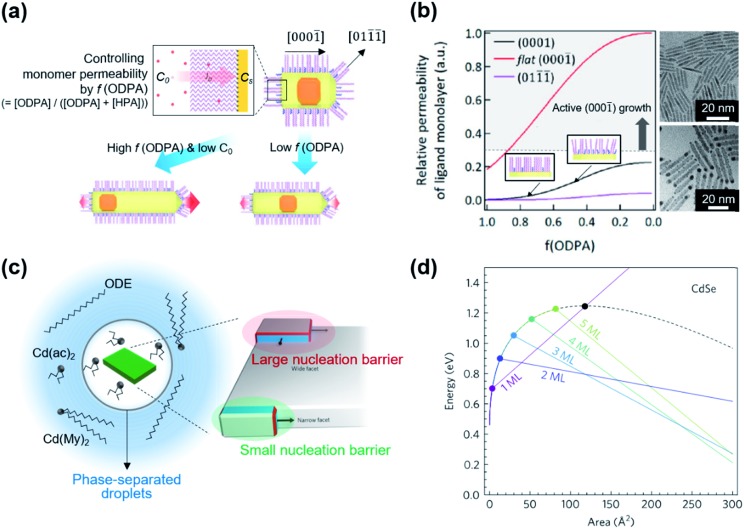
(a) Schematic illustration of the growth mechanism of DiRs governed by monomer permeability by varying f(ODPA). (b) Relative monomer permeability for different facets as a function of f(ODPA), defined as a fraction of ODPA to overall ligands in the reaction mixture (left), and resulting DiR morphologies: a centric case (upper right) and an acentric case [CdSe cores closed to a (0001) facet] (lower right). To visualize core position, NRs are stained with Au. Reproduced with permission from ref. 27. Copyright 2017 American Chemical Society. (c) Schematic illustration on the phase-separated droplets derived by short chain ligands with low solubility in 1-octadecene. The scheme on the right side displays a nucleation of CdSe islands grown on the narrow facet (green shadow) and the wide facet (red shadow). Blue and red facets on the islands depict an increasing surface area resulting from the growth. (d) Total energy of CdSe NPLs as a function of island size on wide and narrow facets for different thicknesses. The black dashed line represents the wide-facet limit. Colored lines display narrow facets for different thicknesses in terms of monolayers (MLs). Cross points stand for nucleation barriers, marked using colored points. Reproduced with permission from ref. 48. Copyright 2017 Nature Publishing Group.

A couple of computational studies revealed that NRs and their heterostructure derivatives composed of metal chalcogenides result from the rapid growth of NCs along the [0001[combining macron]] direction because of weak ligand binding to the (0001[combining macron]) facet for typical reaction conditions.^14^,^43^,^44^ Kim *et al.* suggested that the use of two different surface ligands could extend the control window of nanoscopic facets on dot-in-rods (DiRs).^27^ In the case of low monomer concentration, a facet along the [0001[combining macron]] direction is developed on the (011[combining macron]1[combining macron]) facets (Fig. 3a), which possess comparable surface energy with the (0001) facet but lower monomer permeability as a result of strong ligand–ligand interaction. On the other hand, increase in the monomer concentration develops multiple facets at the tip along the [0001[combining macron]] direction to chemically inactive (011[combining macron]1[combining macron]) and most-active (0001[combining macron]) facets. By employing binary surface ligands (*i.e.*, *n*-hexylphosphonic acid (HPA) and *n*-octadecylphosphonic acid (ODPA)) and perturbing ligand–ligand interaction between hydrocarbon chains, it is possible to control the monomer permeability of each facet over a wide range. Specifically, a ratio of monomer permeability (*P*) between (0001) and (011[combining macron]1[combining macron]) (*P*(0001)/*P*(011[combining macron]1[combining macron])) varies from ∼1 to ∼10 when a fraction of long chain ligand (*f*) decreases from 1 to ∼0.5 and a *P*(0001[combining macron])/*P*(0001) is from >20 to ∼7 when *f* decreases from 1 to ∼0.5 (Fig. 3b, left panel). For embryonic DiRs equipped with dual surface ligands, the versatile structural tunability of CdSe/CdS DiRs is realized, for example, a positional tuning of the CdSe core from the (0001) side to the center of NRs and a dual-diameter NR with a selective occupation of the CdSe core at the thicker phase (TEM images in Fig. 3b).

Even though the crystallographic anisotropy has been one of the key principles to induce anisotropic growth of NCs, recent studies reveal that cadmium chalcogenide NPLs possess an isotropic zinc blende crystal structure.^45^ In these NCs, a basal plane is indexed to be a (100) facet passivated with fatty acids and lateral dimension extends along [001] or [110] directions. Because of the same atomic arrangement of the (100) family, conventional arguments relying on the reactivity difference of various facets are not applicable in this case. And the absence of the mesophase comprising excess ligands excludes the template-assisted growth of NPLs.^46^ Therefore, the growth mechanism behind the NPLs with cubic crystal structures is a subject of ongoing debate. Chen *et al.* explained the growth based on the oriented attachment of seed NCs.^47^ Their experimental assessment reveals that CdSe NPLs grow as a result of the fusion of NCs into 2D intermediates along with (110) facets because of low areal ligand density compared to (100) facets. And the cadmium acetate introduced in the NPL growth plays a role in transforming spherical NCs into quasi-2D intermediates and promoting the oriented attachment.

The controversy on the growth mechanism of NPLs fueled the research efforts to identify conditions that induce 2D anisotropic growth.^48^ In the colloidal growth of NPLs, short chain ligands (*e.g.*, cadmium acetate) play a crucial role in separating the phase of Cd precursors from the solvent. A short chain ligand yields Cd(long carboxylate)_2–*x*_(short carboxylate)_*x*_ with marginal solubility in the reaction medium (typically 1-octadecene) and forms phase-separated droplets. In the absence of the solvent, NPLs can be produced with either long or short carboxylates. Kinetically controlled growth under this concentrated condition promotes the lateral growth of NPLs (Fig. 3c, left). In addition to this experimental finding, they quantify the change of surface energy associated with the nucleation and growth of the CdSe island on the basal plane or at edges of NPLs using density-functional theory. Since the nucleated island on the narrow side did not require additional edge energy in order to span the facet (Fig. 3c, right), the nucleation barrier for the lateral growth (*e.g.*, 0.9 eV for 2 monolayer-thick NPL) is much smaller than that of basal (*e.g.*, 1.25 eV) for the same areal increment (Fig. 3d). This energy difference induces faster growth on thin facets compared to large facets and results in anisotropic growth from isotropic crystal structures.

## Ligands in inter-NC potential control

To capitalize on the unique photophysical properties of individual NCs, their assembly into desired films or clusters can be of paramount significance. For example, NC-based light-emitting diodes require uniform 10s nm-thick NC layers sandwiched between charge transport layers.^49^ On the other hand, thicker NC films or composites with large light absorption are favorable in the case of NC-based light-harvesting devices: *e.g.*, dense 100s nm-thick NC films for photovoltaics^50^/photodetectors,^51^ ∼1000s nm-thick films for ionizing radiation detectors,^52^ or homogeneously dispersed NCs in the millimeter-thick transparent matrix for luminescent solar concentrators.^53^ Turning colloidal NCs in solution into a film with a wide range of thickness and long-range uniformity demands understanding on the various interactions between NCs and their surroundings (*e.g.*, solvent molecules, solution–air or solution–solid interfaces) as well as inter-NC interactions.

Without a surrounding medium and surface ligands, NCs would undergo aggregation during their collision events because of attraction potential between objects, for example, the Derjaguin approximation (∝1/*d*, where *d* is a surface-to-surface distance between NCs) for spherical NCs.^54^ Stable NC dispersions are therefore understood as a presence of repulsive potential provided by surface ligands and surrounding solvent molecules. In a good solvent that is able to solubilize NCs through favorable solvent-ligand interaction, ligand-capped NCs experience an increment of free energy as they are approaching twice the ligand shell thickness.^8^ Excluded solvent molecules around surface ligands suffer from enthalpic destabilization and tend to penetrate back into the ligands, which applies repulsive force between NCs (so-called, osmotic repulsion). Compression of surface ligands is also responsible for the repulsive force to recover bond angles of hydrocarbons (so-called, elastic repulsion) and entropic freedom. And Flory–Huggins theory guides enthalpic and entropic contributions to the free energy of mixing when NCs are in close contact, using *χ* for enthalpic solvent-ligand interaction and overlap integral for entropic penalty involved in the interdigitation of ligand chains.^55^,^56^

**Fig. 4:**
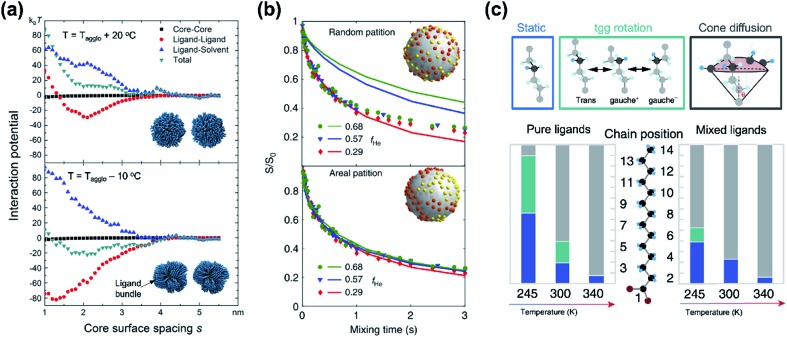
(a) Contribution of attractive (minus sign) and repulsive (plus sign) potentials originated from van der Waals interaction between Au NCs (black square), ligand–ligand (red circle), ligand–solvent (blue upper triangle), and their summation (green lower triangle) at the temperature higher (left) and lower (right) than the aggregation temperature, obtained from constrained molecular simulation. Insets show the conformation of surface ligands at given temperatures. Reproduced with permission from ref. 57. Copyright 2018 American Chemical Society. (b) The ^13^C CODEX decays of CdSe QDs capped with myristate and hexanoate (symbols) and numerical simulation (solid lines) for a fraction of hexanoate (*f*_He_): 0.68 for circles, 0.57 for triangles, and 0.29 for diamonds. The upper (lower) panel shows a comparison of experimental data with a scenario of random distribution (partition distribution). Insets illustrates the spatial partitioning of hexanoate (yellow) and myristate (red) on QDs. (c) Potential conformational mode of myristate ligands and their relative population in QDs with myristate-only ligands (left) and with a mixture of myristate and hexanoate (right). Stationary –CH_2_–, tgg rotation, and cone diffusion are represented as blue, green, and grey colors, respectively. Reproduced with permission from ref. 58. Copyright 2019 Nature publishing group.

While classical colloid theory provides qualitative guidelines to choose good solvent–ligand pairs for NC dispersion, temperature or size dependent aggregation behaviors of nanometer-sized NCs that are important behaviors in practical areas demand more profound understanding on the molecular scale. A computational study on the aggregation behavior of 1-hexadecanethiol-capped Au NCs in decane by Kister *et al.* provides an excellent starting point to discuss inter-NC interactions.^57^ For convenience, here, we address the relative strength of attractive and repulsive potentials between NCs in terms of *k*_B_T (*ca.* 26 meV at room temperature). According to their molecular simulation and also other experimental investigation,^20^ it is widely accepted that ligands form densely packed bundles by van der Waals interaction between hydrocarbon chains and undergo order-disorder transition as temperature is elevated (see insets in Fig. 4a). The constrained molecular dynamics simulation for Au NCs with a diameter of 4.6 nm shows that the ligand–ligand and ligand–solvent interactions are scaled as tens of *k*_B_T per NC–NC pair depending on their distance while van der Waals attraction between cores, merely scaled as ∼*k*_B_T. Summation of all potential terms scaled as 10–20 *k*_B_T per NC–NC pair and the ligand–ligand interaction term mainly determines whether NCs are aggregated (Fig. 4a). In addition, the energetics based on molecular simulation exhibit a good accordance with NC aggregation temperatures for small and moderate NCs while the enthalpic and entropic energies obtained by Flory–Huggins theory are unable to explain size-dependent aggregation behaviors. They attributed the attraction potential between NCs to the interlocking of ligand bundles as well as the interdigitation of ligand shells.

Kister *et al.* pointed out that the classical approach based on Flory–Huggins theory underestimates enthalpic and entropic interaction of surface ligands with two NCs approaching.^57^ In an independent study, Yang *et al.* also accounted for the lack of appropriate interpretation for enthalpic and entropic changes of closely packed NCs and provided additional energetic contribution by surface ligands in NC dispersion.^20^ They divide the dissolution of NCs in two steps, (i) melting of ligand bundles and interdigitated ligands on NCs; and (ii) mixing with solvent molecules. While enthalpic and entropic gain (Δ_mix_*G* = Δ_mix_*H* – *T*Δ_mix_*S*) resulting from the mixing is merely ∼*k*_B_T, assuming NCs as hard spheres, the enthalpic cost for melting hydrocarbons of ligand shells is tens of *k*_B_T. Peng and coworkers proposed a concept ‘entropic ligands’ for boosting the solubility of NCs, which allows for governing the total Gibbs free energy of NC dissolution by entropy gain of ligand chains, not by melting enthalpy of ligand shells.^58^,^59^ They demonstrated that mixtures of two linear hydrocarbons with different lengths (*e.g.*, myristic acid and hexanoic acid) perturb the ligand–ligand interaction by intervening short branches between long hydrocarbon chains. Such disturbed inter-ligand interaction liberates surface ligands from ligand bundles induced by van der Waals packing and results in ligand shells being melted for a wide range of temperature, which boosts the solubility of NCs up to ∼6 orders of magnitude (*i.e.*, 100s mg mL^–1^).

Nuclear magnetic resonance (NMR) spectroscopy has been used to quantify the average areal density of surface ligands,^60^ progression of ligand exchange reaction,^61^ or adsorption/desorption dynamics of ligands.^62^ In many cases, original or incoming ligands have a distinguishable marker in a hydrocarbon chain (*e.g.*, methine proton) that locates at downfield (*ca.* 5.2–5.7 ppm) compared to methyl protons (*ca.* 0.8–2 ppm). For example, Balan *et al.* quantified the amount of ligands as well as the ligand packing density of ODPA and OA co-passivated CdSe/CdS QDs using ^1^H NMR: the average fraction of OA and ODPA is quantified by contrasting areal ratios between the methine protons of OA and methyl protons of ODPA.^63^

Recent advances in the characterization of surface ligands using solid state NMR (SSNMR) in combination with numerical simulation enabled quantitative analysis of the dynamic behavior and spatial distribution of ligands at the molecular level. Pang *et al.* analyzed the spatial positioning of entropic ligands composed of myristate and hexanoate by probing ^13^C centerband-only detection of exchange (CODEX) SSNMR.^58^ The CODEX decay indicates the ^13^C chemical shift exchange of ^13^C-labelled myristates during NMR mixing times. Indistinguishable CODEX decay as a function of a hexanoate fraction suggests that each ligand occupies segregated surface domains rather than random sites, which is in agreement with numerical simulation (Fig. 4b). In addition to verifying spatial distribution of ligands, dynamic modes of hydrocarbon segments of ligands can also be quantified by ^2^H NMR. ^2^H NMR detects reorientation modes of hydrocarbon segments, namely, static –CH_2_–, trans–gauche^+^–gauche^–^ (tgg) rotation, and most flexible cone diffusion (Fig. 4c, top). Deconvolution of ^2^H NMR patterns reveals that the presence of hexanoate penetrated in myristate ligands promotes the cone diffusion of nearly entire methylene units in the chain in a wide range of temperature, which leads to enhanced conformational freedom of ligand chains (Fig. 4c, bottom).

While most spherical NCs exhibit isotropic optical and electrical characteristics, anisotropic NCs such as NRs or NPLs demonstrate unique dimensionality-driven properties such as polarized absorption and emission^24^,^64^ or orientation-dependent energy transfer rate.^65^ In addition, such properties are particularly beneficial for practical applications. For instance, in-plane orientation of NRs has recently attracted keen attention in the field of displays because of enhanced light out-coupling from aligned NRs.^66^,^67^

Long-range ordering of anisotropic NCs (from several to 10s inch in terms of diagonal length) represents a significant hurdle in the way of using the anisotropic NCs in display applications. Unlike spherical NCs with a rotational and plane symmetry, anisotropic NCs require specific orientations to form closely packed, ordered structures. For instance, emissive NRs for displays need to be assembled in the in-plane direction since the transition dipole of NRs in out-of-plane direction impedes the light extraction from substrates.^68^ However, preferential side-to-side van der Waals attraction compared to that of end-to-end tends to form randomly oriented NR bundles during the assembly. Such an observation evidently suggests the requirement of additional tools to manipulate the NC–NC interaction during the assembly in a directional manner.

As we discussed above, the van der Waals interaction between NCs is merely scaled as ∼*k*_B_T while this interaction can be intensified by shape and orientation of anisotropic NCs. While several ordered NC arrays can be accomplished purely by van der Waals attraction at liquid–air^69^ or liquid–liquid interfaces,^70^ to provide sufficient driving force surely exceeding *k*_B_T, many research utilizes the depletion attraction^71^ by introducing a small amount of depletant. The depletion attraction originates from the entropy gain by NC aggregates in the presence of depletants. Overlap of the excluded volume of NCs, a depletant-inaccessible region defined by the radius of hard depletant spheres, increases the free volume allowed for depletants, and consequently, increases the entropy of the entire system as the NCs form aggregates. Since the fundamental principle was established by Asakura and Oosawa for hard spheres and non-interacting depletants,^72^ the depletion attraction has been widely employed in assembly of NCs.^73^–^75^

Depletion attraction of several-nm sized NCs is conceptually the same as the conventional description but requires a molecular picture to explain its driving force. The excluded volume of NCs can be translated as the volume of ligand shells with a depth of <1 nm for typical oleate ligands. Accessibility of depletant molecules is limited by entropic penalty, if the depletant is athermal to ligands. Such an illustration naturally deduces that the surface ligands can become one of the molecular tools to manipulate the extent of depletion attraction and corresponding NC assemblies.

**Fig. 5:**
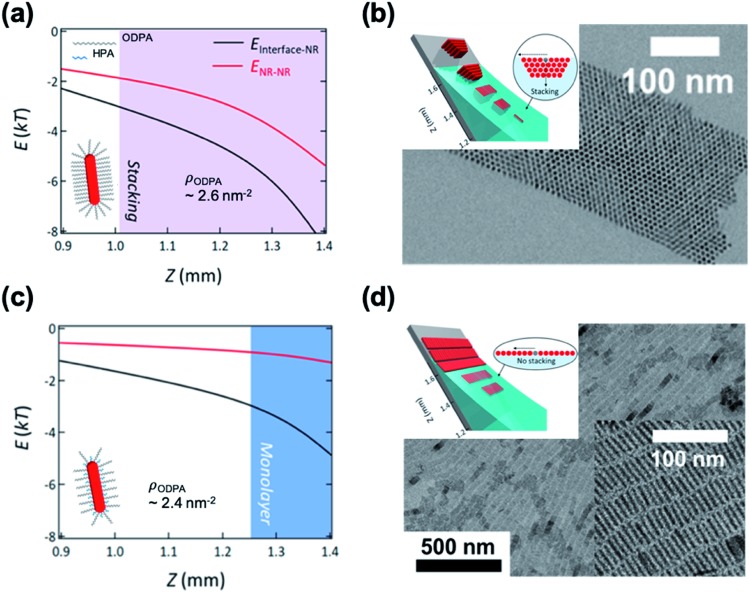
Position (*Z*) dependent interaction potentials between NRs (black) and NRs – interface (red) during dip coating for varied ligand densities of ODPA (*ρ*_ODPA_) (a and c) and corresponding self-assembled structures (b and d): *ρ*_ODPA_ = 2.6 nm^–2^ (a and b) and *ρ*_ODPA_ = 2.4 nm^–2^ (c and d). Decrease in *ρ*_ODPA_ lowers the interaction potential of DiRs with counterparts and minimal NR–NR interaction potential yields the smectic DiR monolayer. Reproduced with permission from ref. 73. Copyright 2019 American Chemical Society.

Kim *et al.* demonstrated the influence of surface ligands of NCs in the context of depletion attraction and corresponding self-assembly during the dip coating process.^73^ For CdSe/CdS DiRs capped with ODPA, the surface ligand density of ODPA (*ρ*_ODPA_) was modified by partial ligand exchange with HPA (see cartoons in Fig. 5). On adjusting *ρ*_ODPA_ from 2.6 nm^–2^ to 2.4 nm^–2^, they observed the development of assembled structures from vertically aligned DiR bundles (Fig. 5b) to multilayered stacks to smectic monolayers (Fig. 5d) with oleic acid as a depletant. In this system, the excluded volume originating from the ligand shell decreases as the *ρ*_ODPA_ decreases because perturbation of ligand packing by HPA increases the accessibility of depletants into the ligand shells. This is supported by a simple precipitation test for varied *ρ*_ODPA_ and a concentration of depletant in which reduced *ρ*_ODPA_ sustained colloidal stability for a wide range of concentrations of oleic acid. In a similar manner, depletion force driving DiRs to the interface is also weakened with decreasing *ρ*_ODPA_. Calculation on inter-NCs and NC-interface interaction potentials revealed that the decrease in *ρ*_ODPA_ lowers the extent of depletion attraction for both cases. Relative attraction force at the liquid–air interface during the dip coating determines the favorable arrangement of DiRs (Fig. 5a and c). The smectic DiR monolayer is attributed to the weakened DiR–DiR attraction by depletion force that places DiRs to the liquid–air interface, not DiR bundling. Their finding suggests that, even weak interaction potential between NCs competing with *k*_B_T, tailoring chemical composition of surface ligands can be one of the influential factors to manipulate the inter-NC interaction.

## Summary and Outlook

Research on colloidal semiconductor NCs has expanded from the academic to industrial realm, as the arrested precipitation approach has enabled a facile and scalable synthesis protocol for NCs. In the colloidal growth, ligands hold unequivocally important standing. While seminal studies on the growth of CdSe NCs rightfully place an emphasis on the decomposition kinetics of precursors at high temperatures,^5^,^6^ the ligands that adsorb to the NC surface are also responsible for successful exploitation of the colloidal synthesis. The past three decades have witnessed the remarkable progress in experimental techniques in which composition and configurations of the NC surface provided an opportunity space for more quantitative studies for ligands. For instance, a combination of NMR and X-ray photoelectron spectroscopy allows us to clarify the binding mode of surface ligands and their areal density. Z-contrast imaging of high resolution dark field imaging allows us to visualize facet development of NCs and three-dimensional structures directly. Advanced solid-state ^13^C NMR and ^2^H NMR techniques even reveal dynamic natures of surface bound ligands as well as spatial distribution on the NC surface for complicated mixed ligand systems.^58^ Combination of all those techniques is able to clarify the structure of semiconductor NCs down to the atomic scale and be greatly helpful to interpret development of NCs guided by multiple ligands.

Based on the understanding of the nature of ligand-NC interactions, the research progress has elevated the possibility of atomic-scale precision in size and shape control. In a multiple ligand approach, one can take advantage of different types of ligands to design NCs under better control. The combination of ligands with different surface binding modes or conformations enables the isotropic NCs to develop selective facets. In addition, the chain length of ligands and their packing density can serve as additional operation parameters for the controlled growth of anisotropic NCs such as NRs and NPLs. The ligand layer on NCs serves as a steric barrier for monomer permeation and inter-NC fusion. The combination of short- and long-chain ligands enables us to tailor the chemical reaction in a controlled manner.

The organization of materials, from the atomic to macroscopic scale, has long been an area of intense study. The progress in X-ray scattering techniques helps deduce the structure of assembled structures, leading to a greater understanding of how ensemble properties depend on packing of the constituent building blocks.^76^ The unique size-dependent properties of semiconductor NCs has sparked interest in the formation of ordered NC thin films, because many applications would require controlled assembly of NCs into thin films. Ensemble properties result from relative orientation of NCs within the films, making the inter-NC interactions all the more important to understand. Colloidal NCs represent a different class of colloid featuring hard cores with soft surface layers constituted with organic ligands; therefore, their assembly requires soft-sphere assumptions rather than classical hard-sphere models.^77^ The soft-sphere interactions between NCs can be approximated by contributions from van der Waals attraction between hard cores, steric repulsion from the capping ligands, and enthalpic interaction of the ligands with surroundings. Recent studies suggested that ligand packing and their dynamic nature alter the permeation of solvents, reactants, or depletants, alluding to exacting control over long-range organization. In particular, the use of multiple ligands provides an extra handle on the solubility of NCs by minimizing the contribution of mixing enthalpy associated with the ligand packing, as the random placement or multiple ligands suppress the enthalpic gain induced by ligand packing and/or ligand bundle interdigitation. This multiple ligand approach works in assembly of CdSe NRs to a greater extent, where depletion attraction can be precisely tuned by the areal density of surface ligands.

While our discussion in this review gravitates to semiconductor NCs, the call for the sophisticated NC design is also essential in metal or metal oxide NCs, guided by employing multiple ligands. For instance, there is already a lot of work demonstrating facet-dependent catalytic activity and its importance, such as CO_2_ reduction on TiO_2_,^78^ formic acid oxidation on Pd,^79^ or benzyl alcohol oxidation on WO_3_ 80 that is enhanced by elongated or polyhedral NCs with specific facets. And such morphology tuning is enabled by employing multiple ligands that preferentially bind to specific surface configuration.^81^,^82^ We believe that the multiple ligand-based wet chemistry will be generalized in future research studies utilizing finely engineered colloidal NCs for various application fields.

Despite the wealth of information and experience in the study of organic ligands in semiconductor NCs, there exist many questions that remain to be addressed. For example, organic surface ligands have low conductivity, which makes the charge transport in NC films hampered by inefficient hopping between NCs. While the issue on poor conductivity has been partially mitigated in the fields of NC-based photovoltaics^83^ and field-effect transistors^84^,^85^ by replacing original organic ligands with short inorganic ligands (*e.g.*, molecular metal chalcogenides^86^ and metal-free anionic ligands^87^), resulting films are mostly glassy owing to the lack of NC–NC interaction control. To boost high performance NC-based electronics featuring high conductivity and low resistive-capacitive delay, we believe that the ordering of semiconductor NCs capped with inorganic ligands needs to be improved and multiple ligands can play a pivotal role in controlling interactions between inorganic ligand capped NCs.

The use of colloidal semiconductor NCs in light-emitting devices has escalated the expectation of their use in next-generation light sources with superior brightness, color purity, and energy efficiency. In particular, anisotropic NCs with well-defined dipole orientation can also be an interesting class of light-emitting materials. Yet, despite the tremendous progress in the growth and assembly of anisotropic NCs, there remain several questions to address in terms of atomic-precision growth control and ordered array of individual NCs. For instance, the surface configuration of edge facets in CdSe NPLs is a subject of ongoing discussion.^45^ Comprehensive understanding of the surface and ligand configuration for edge facets in NPLs would help realize complex 2D morphologies by achieving atomic precision growth between edge facets such as (100), (110), and (111). As for the assembly of anisotropic NCs, long-range and directional ordering holds delicate control and detailed understanding of ligand dynamics more accountable.^88^,^89^

## Conflicts of interest

There are no conflicts to declare.
